# Identification of Mutation Landscape and Immune Cell Component for Liver Hepatocellular Carcinoma Highlights Potential Therapeutic Targets and Prognostic Markers

**DOI:** 10.3389/fgene.2021.737965

**Published:** 2021-09-16

**Authors:** Hengzhen Wang, Wenjing Jiang, Haijun Wang, Zheng Wei, Hali Li, Haichao Yan, Peng Han

**Affiliations:** Department of General Surgery, The First Affiliated Hospital of Harbin Medical University, Harbin, China

**Keywords:** liver hepatocellular carcinoma, somatic mutation, RNA-sequencing, genome variation, precision medicine

## Abstract

Liver hepatocellular carcinoma (LIHC) is a primary malignancy, and there is a lack of effective treatment for advanced patients. Although numerous studies exist to reveal the carcinogenic mechanism of LIHC, few studies have integrated multi-omics data to systematically analyze pathogenesis and reveal potential therapeutic targets. Here, we integrated genomic variation data and RNA-seq profiles obtained by high-throughput sequencing to define high- and low-genomic instability samples. The mutational landscape was reported, and the advanced patients of LIHC were characterized by high-genomic instability. We found that the tumor microenvironment underwent metabolic reprograming driven by mutations accumulate to satisfy tumor proliferation and invasion. Further, the co-expression network identifies three mutant long non-coding RNAs as potential therapeutic targets, which can promote tumor progression by participating in specific carcinogenic mechanisms. Then, five potential prognostic markers (*RP11-502I4.3, SPINK5, CHRM3, SLC5A12*, and *RP11-467L13.7*) were identified by examining the association of genes and patient survival. By characterizing the immune landscape of LIHC, loss of immunogenicity was revealed as a key factor of immune checkpoint suppression. Macrophages were found to be significantly associated with patient risk scores, and high levels of macrophages accelerated patient mortality. In summary, the mutation-driven mechanism and immune landscape of LIHC revealed by this study will serve precision medicine.

## Introduction

Liver hepatocellular carcinoma (LIHC) is the most common primary malignancy of the liver and the third leading cause of cancer-related death worldwide (Bosch et al., 1999; Bray et al., 2018). Of these, liver cancer is the second leading cause of cancer-related death in LIHC, accounting for approximately 90% of all primary liver cancer cases (Llovet et al., 2016). Studies have found that fat accumulation of liver can lead to non-alcoholic steatohepatitis, cirrhosis, liver failure, and LIHC (Kim et al., 2021). Treatments for LIHC include hepatectomy, liver transplant, chemotherapy, and molecular targeted therapy. However, clinical treatment results show that these treatments are not effective for LIHC patients (Heimbach et al., 2018). Therefore, there is an urgent need for the identification of new therapeutic targets for the development of new drugs.

Somatic variations, including copy number variations (CNVs) and point mutations, are considered to be the driving event for the occurrence and development of cancer. In recent years, researchers mainly focused on key mutated genes and their mutational characteristics (Zhang et al., 2021). However, the integration of mutagenomics with other omics data is more powerful in revealing the pathogenesis of patients and potential therapeutic targets (Fujimoto et al., 2016). With the development of next-generation sequencing, multiple somatic variations have been discovered. Especially, accumulated studies have demonstrated that somatic variations, such as single-nucleotide variations and CNVs, could contribute to tumorigenesis (Wang et al., 2020) and used to infer individual medications based on the RNA interaction network (Zhang et al., 2018). Based on the notion that the instability of the genome is related to age (Chatsirisupachai et al., 2021), it is crucial to investigate the relationship between the stability of the genome and the physiological mechanism of the patient. More recently, large-scale biomedical data, including multidimensional molecular profiles of tumor samples of LIHC generated by The Cancer Genome Atlas (TCGA; Tomczak et al., 2015) project, provide opportunities to uncover mutation-driven potential therapeutic targets and potential prognostic markers for liver cancer.

Over the past decade, the immune microenvironment has been a popular area of cancer biology research in relation to therapeutic targets. The immune microenvironment is composed of a variety of lymphocytes, such as T cells, B cells, and macrophages. Previous studies have shown that the composition of immune cells is closely related to tumor proliferation and metastasis. For example, CD8+ T cells show strong cytotoxic activity on tumor cells and have a strong inhibitory effect on tumor progression (Seo et al., 2018). Macrophage polarization plays a key role in subverting adaptive immunity and promoting tumor progression (Mantovani et al., 2002). The development of the immune cell fraction algorithm (Newman et al., 2015) for bulk RNA-seq data provides convenience for investigating the relationship between specific immune cell content and tumor progression.

In the current study, we integrated and analyzed the somatic mutations, CNVs data, and RNA-seq of LIHC collected from the TCGA database. The mutation landscape of LIHC and the metabolic features driven by mutations were revealed. Our work highlights potential therapeutic targets, potential prognostic markers, and the role of macrophages in tumor progression. These results promote the understanding of pathogenesis and provide a basis for the treatment of LIHC.

## Materials and Methods

### Data Collection

The CNV data, somatic mutation data, clinical information, and RNA-seq profiles of LIHC collected by TCGA (Tomczak et al., 2015) were downloaded from UCSC Xena browser.^1^ Metabolic pathway and hallmark gene sets that will be used for metabolic feature analysis and enrichment analysis of carcinogenic functions for LIHC were collected from the Molecular Signatures Database (Liberzon et al., 2015).^2^ Moreover, the annotation data of GRCh38 v29 for long-noncoding RNA (lncRNA) were collected from GENCODE (Frankish et al., 2019).^3^ The signature matrix of 22 immune cell types was collected from the previous studies (Newman et al., 2015) for the analysis of immune cell invasion of tumor samples.

### Processing of Mutation Data

We first counted the distribution of mutation sites on the human genome, including mRNA, lncRNA, and transcription start site, as well as the distribution of various types of mutation, including missense and nonsense mutation on the chromosome. Further, the R package maftools (version 2.8.0; Mayakonda et al., 2018) was used for the statistical and visualization of mutation form, mutation frequency, and mutational correlation between genes, which provides great convenience for the research of mutation data and the reveal of characteristics. The number of mutations in each tumor sample was calculated and used to link the CNV data. We downloaded the GDC GISTIC copy number dataset from the UCSC Xena browser, which is derived from focal copy number estimates, and the positions of the variant sequence corresponding to the genes. Both gene amplification and deletion events are thought to increase genome instability. By integrating the mutation information and gene copy number information of patient cohort, we defined the top 20% of patients with copy number amplitude and mutation load as high-genomic instability group, the bottom 20% of patients with copy number amplitude and mutation load as low-genomic instability group, and the remaining patients as median/unknown-genomic instability group.

### Gene Set Enrichment Analysis

Considering that there were multiple zero values in the gene expression matrix, we control the number of genes by requiring effective genes to be expressed in at least 10% of tumor samples. Based on the previously defined high/low-genomic instability samples, the rank sum test was used to identify genes that are significantly differentially expressed in the high/low-genomic instability samples. The cutoff of value of *p* is set to 0.01. For these significantly differentially expressed genes (DEGs), the genes were sorted using the logarithmic fold change as the weight and combined with the hallmark gene set to be used for gene set enrichment analysis (GSEA; Subramanian et al., 2005) by R package fgsea (version 1.1.0). We set the value of *p* to <0.05 to screen out carcinogenic functions that are significantly enriched on DEGs.

### Calculation of Metabolic Pathway Activity

Gene set variation analysis (GSVA; Hanzelmann et al., 2013), which is an unsupervised manner to estimate changes in pathway activity over a sample population, was used to calculate the metabolic activity of each tumor sample by R package GSVA (version 1.32.0). We set the number of genes in the gene set used for functional enrichment to be at least 10 and not more than 500. The rank sum test and fold change algorithm were also used to calculate the variation of metabolic pathway activity between high and low-genomic instability samples. Metabolic pathways with a value of *p*<0.01 were considered to be affected by mutations, and reprogramming has occurred.

### Construction of Co-expression Network Mediated by Mutant lncRNA

We extracted lncRNA from DEGs which differentially expressed between high- and low-genomic instability samples based on lncRNA annotation data obtained from GENCODE. By combining somatic mutation and CNV data, we identified lncRNAs that were mutated in tumor samples and differentially expressed in the high-genomic instability group, defined as mutation-driven lncRNA (Md-lncRNA). Next, the Pearson correlation algorithm (Bishara and Hittner, 2012) is used to calculate the correlation between Md-lncRNAs and other DEGs, which was *performed by cor.test function of R. We have defined that gene pairs with* value of *p*<0.01 and | R |>0.3 have significant correlation in expression and are co-expressed with each other (van Dam et al., 2018). For these co-expressed genes, cytoscape (Shannon et al., 2003) was used to plot the co-expression network, and Network Analyzer tool was used to calculate the topological properties of the network.

### Identification of Potential Prognostic Markers

The genes in the co-expression network mediated by Md-lncRNAs were used as candidate markers. We first used univariate COX regression and lasso regression (Alhamzawi and Ali, 2018) to screen genes that significantly associated with overall survival (OS) of LIHC patients (the cutoff of value of *p* was 0.05). Next, we randomly selected 60% of all samples as the training set and the remaining as the test set. The training set was used to construct a multivariate COX regression model (Fisher and Lin, 1999). We retained the genes passing the test of multivariate COX regression as potential prognostic markers and establish nomogram to predict the OS of LIHC. The reliability of the prediction model was validated by the receiver operating working characteristic curve (ROC), and the area under curve (AUC) also was calculated. The calibration curve was used to evaluate the predictive power of nomograph for survival risk.

### Survival Analysis

The risk score for each patient was calculated according to the linear combination of expression values weighted by the coefficient from the multivariate Cox regression analysis:


$$Riskscore\;\left(i\right)=\sum_{k=1}^{n}\beta_{k}*e_{ki}$$

where *n* denotes the number of prognosis markers (*n*=5), β was the coefficient of multivariate Cox regression analysis, and $e_{ki}$ was the expression level of kth prognosis-related gene expression of patient i. Further, the samples of train set and test set were, respectively, divided into high- and low-risk categories based on the median risk score calculated by risk score model, and Kaplan–Meier algorithm (Ranstam and Cook, 2017) was used to compare whether the survival data of the two categories are different and bilateral log-rank test was used to validate the significance of the difference.

### Calculation of Immune Cell Fraction

Based on the feature matrix of 22 immune cells obtained from previous studies, the CIBERSORTx tool^4^ (Newman et al., 2015, 2019) was used to analyze tumor-infiltrating immune cells. CIBERSORTx is a method to characterize the cell composition of complex tissues from the gene expression profile. The parameter perms that the number of permutations when calculating the value of *p* was set to 1,000, and QN was set to TRUE to perform quantile normalization. In order to see more group differences in other cell types other than plasma cells, we further transformed the original cell components into a log ratio of log (the fraction of plasma-cell +1e-3)/log (the fraction of immune-cell +1e-3) (He et al., 2021).

### Statistical Analysis

All statistical analyses and graph generation were performed in R (version 4.0.2) and GEPIA (version 2.0).^5^

## Result

### A Global View of Mutations for Liver Hepatocellular Carcinoma

**Figure 1:**
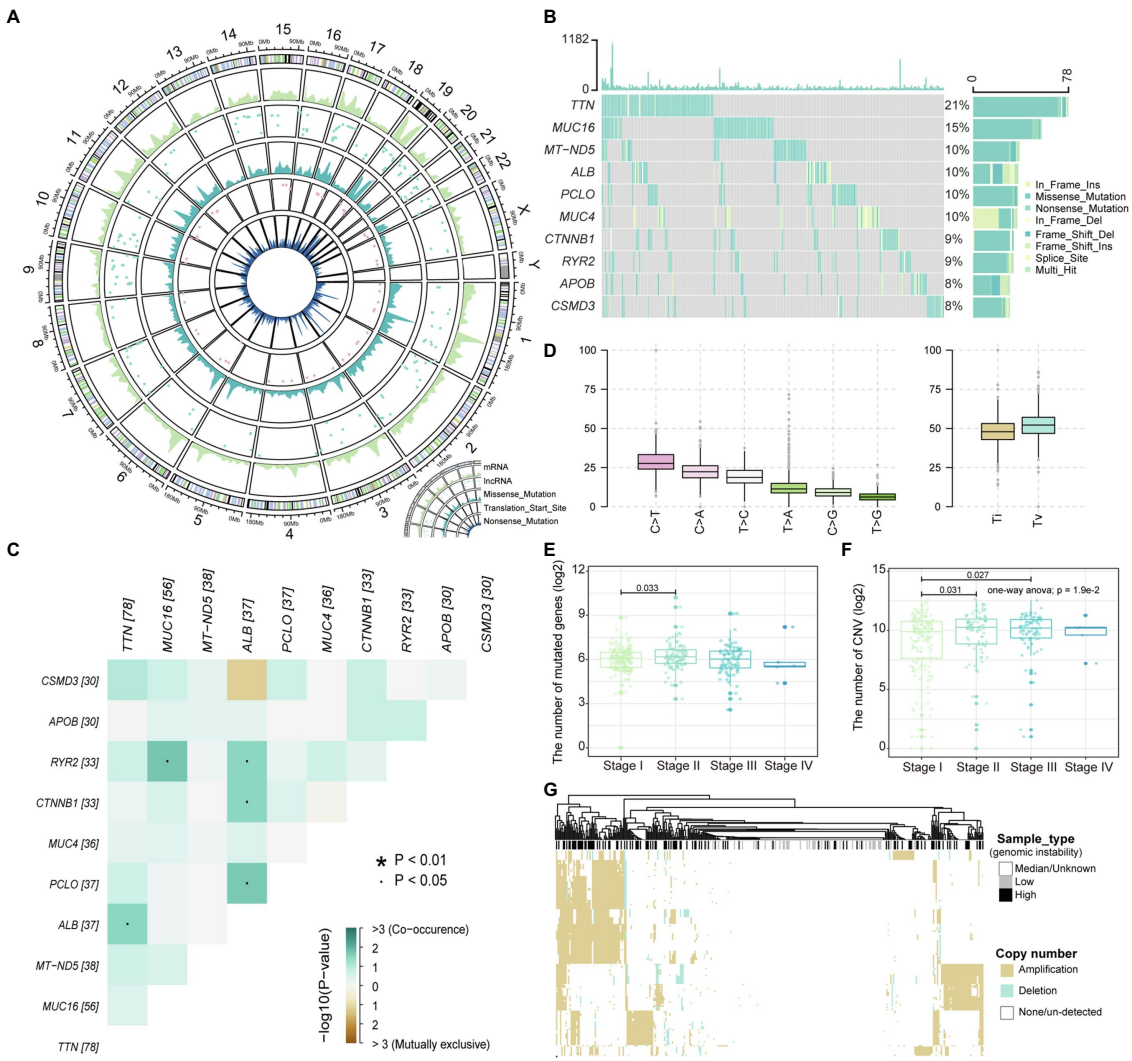
The landscape of liver hepatocellular carcinoma (LIHC) somatic variations. **(A)** The density distribution of somatic mutations on chromosomes. The four-layer circle plot shows the density distribution of nonsense mutations, mutations of transcription start site, missense mutations, mutations of long-noncoding RNA (lncRNA), and mutations of mRNA on chromosomes from inside to outside. **(B)** The waterfall plot shows the top 10 genes in terms of mutation frequency by sample. The mutation type of each gene in each sample is marked. **(C)** Mutation correlation heatmap of the top 10 high-frequency mutated genes. Locations with significant correlations are marked by stars. **(D)** Boxplot shows the frequency of base substitutions including transversion and transition. **(E,F)** The relationship between the number of mutated and copy number variation (CNV) genes in each sample and the stage are displayed with boxplot. The number of mutated and CNV genes is logarithmized, and the rank sum test is used to assess differences between groups. **(G)** The copy number amplitudes of tumor samples are presented in heat map. Column labels show sample types, including high/low-genomic instability and median.

Malignant mutations in the genome are the underlying cause of tumor development and progression. The identification of mutation characteristics is essential for the exploration of pathogenesis. We have first used maftool to evaluate mutation profiles of LIHC in the TCGA database collection for which somatic mutation data were available. A total of 44,847 somatic mutation sites in 375 samples were included in this study. We counted the distribution of somatic mutations on the genome and found that somatic mutations are significantly enriched in specific regions of chromosomes 1, 11, 17, and 19 (Figure 1A), indicating that the global mutations of LIHC have preference for location. Compared with transcripts (mRNA) of protein-coding genes, fewer somatic mutations occurred in lncRNAs; Figure 1A), indicating that somatic mutations were more likely to directly affect the expression of protein-coding genes and the structure of proteins. However, few mutations in non-coding genes were still the main determinants of human diseases (Maurano et al., 2012). Mutations in the transcription start site will regulate gene expression levels before transcription, which rarely occur on autosomes 4 and 13 in LIHC. Point mutations, including missense and nonsense mutations, are an important part of somatic variations, and LIHC shows the dominant position of missense mutations (Figure 1A and Supplementary Figures S1A,B). Further, we counted the frequency of mutations in each gene, and the top 10 mutated genes were identified (Figure 1B). *TTN*, the gene considered to be most frequently mutated in the pan-cancer cohort (Oh et al., 2020), tended to have missense mutations in LIHC. The content of albumin encoded by *ALB* has been confirmed to be closely related to tumor development and patient prognosis (Li et al., 2018). We found that there was a significant mutational correlation between the genes *TNN* and *ALB* (Figure 1C), which indicates that *TNN* and *ALB* may play a synergistic role in LIHC. We found that almost a quarter of point mutations in LIHC patients were C>T substitutions (Figure 1D; Supplementary Figure S1C). Transitions and transversions, as the two types of DNA base transformations, account for similar proportions in the entire LIHC point mutation (Figure 1D). Mutations of transversions, which account for a relatively high proportion, may be a key factor in liver tissue degradation. By combining the mutation with the patient’s clinical information, we found that patients of stage II have a higher number of mutated genes compared to stage I (Figure 1E), which indicates that the accumulation of mutations appears as the stage increases. We introduced copy number data of LIHC patients, further confirming that advanced patients have a higher accumulation of variation and genomic instability (Figure 1F). Next, we defined high and low-genomic instability samples by integrating somatic mutation and copy number data. We found that the high-genomic instability samples in LIHC have overall gene amplification (Figure 1G). Taken together, all these revealed the mutational features of LIHC.

### Metabolic Reprogramming Affected by Accumulation of Mutations

**Figure 2:**
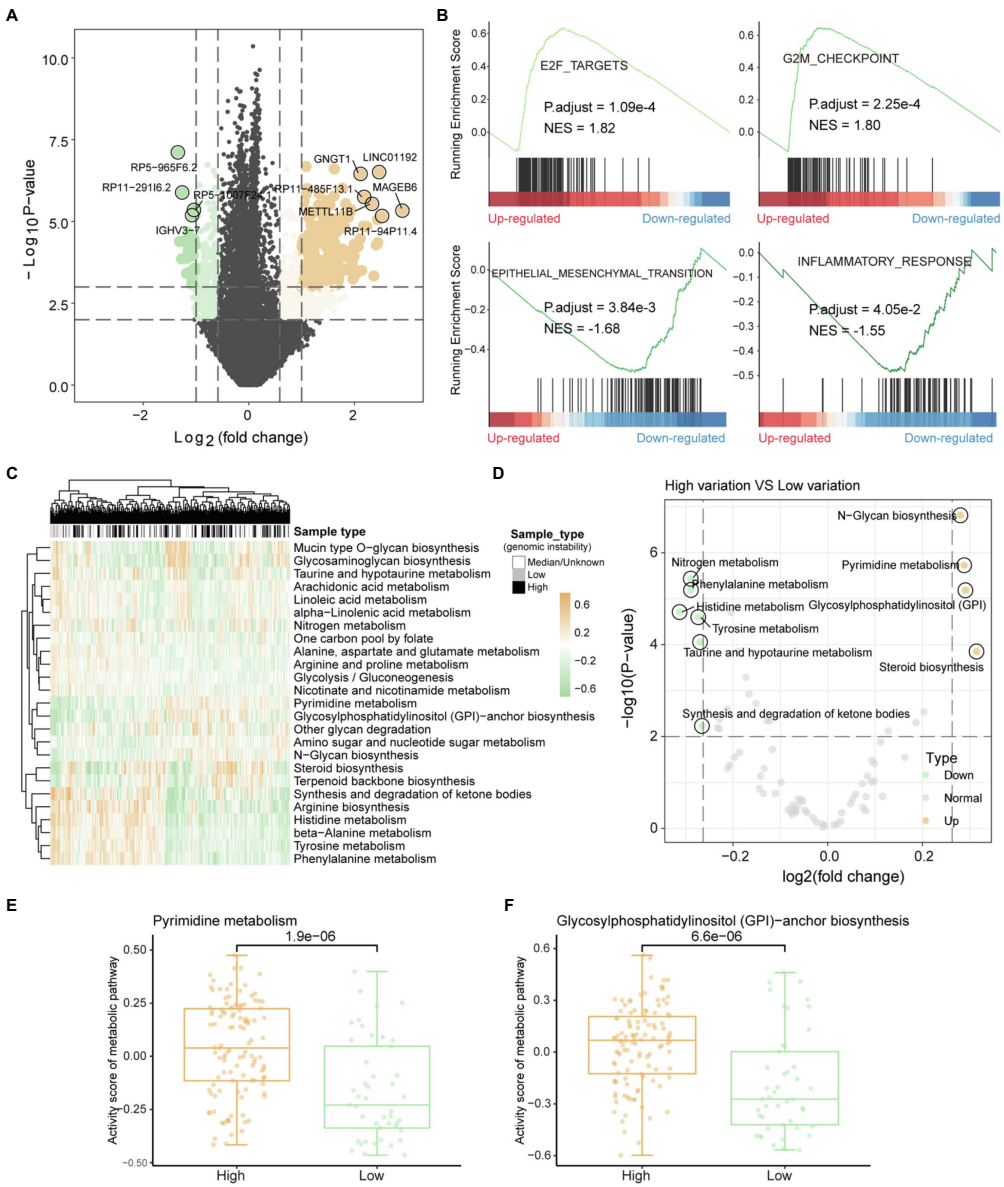
Metabolic remodeling based on genome instability. **(A)** The results of the differential gene expression analysis between high-genomic instability and low-genomic instability samples are shown by the volcano graph. Grey dots represent non-differentially expressed genes, yellow dots indicate genes upregulated in high-genomic instability samples, and the green dots mean the opposite. **(B)** gene set enrichment analysis results of differentially expressed genes. Normalized enrichment score and corrected value of *p* are calculated. **(C)** The enrichment scores of tumor samples in each metabolic pathway calculated by GSVA are displayed by heat map. Column labels show sample types, including high-/low-genomic instability and median. **(D)** Analysis of the difference of metabolic pathway activity scores between high-genomic instability and low-genomic instability samples. **(E,F)** Comparison of pyrimidine synthesis and glycosylphosphatidylinositol-anchor biosynthesis pathway activity among high-/low-genomic instability samples. The rank sum test is used to calculate the significance.

Genome variation can indirectly affect the metabolic efficiency of organisms by regulating gene expression. The rank sum test was used to identify genes that are significantly DEGs between high and low-genomic instability samples. We identified 6,438 DEGs (value of *p*<0.01), including 2,981 upregulated genes and 3,457 downregulated genes (Figure 2A). After GSEA, we identified four carcinogenic functional pathways that are significantly enriched in DEGs (value of *p*<0.05). We found that the E2F pathway, which forms with CDK-RB driving cell cycle progression (Kent and Leone, 2019), is significantly enriched in upregulated DEGs (Figure 2B), indicating that the cell cycle is severely affected by the accumulation of mutations. The G2/M checkpoint can effectively detect the genome and prevent cells from entering mitosis (Anand et al., 2020), which dysfunction may be a key factor in the accumulation of mutations in high-genomic instability samples. We found that the inflammatory response was significantly enriched in the downregulated DEGs (Figure 2B), which may be due to the accumulation of mutations that caused the weakening or loss of tumor tissue immunogenicity (Capietto et al., 2020). All these indicate that the resistance of some patients with advanced liver cancer to immune targeted therapy (Zongyi and Xiaowu, 2020) may be due to the loss of immunogenicity caused by the excessive accumulation of mutations.

Metabolic reprograming affected by mutations was the basis for satisfying tumor proliferation and invasion. Gene set variation analysis (GSVA) was used to evaluate the metabolic activity of each tumor sample. By clustering the metabolic pathway activity score matrix, we found that there are obvious differences in metabolic function between the high and low-genomic instability samples (Figure 2C). Compared with low-genomic instability samples, high-genomic instability samples had higher pyrimidine synthesis activity (Figures 2D,E). Previous studies have shown that inhibiting the metabolic activity of pyrimidine synthesis can effectively reduce the carcinogenic ability of tumors (Wang et al., 2019), which indicates that pyrimidine driver mutations that trigger pyrimidine anabolic remodeling can be used as therapeutic targets for patients with advanced liver cancer. We found that the activity of glycosylphosphatidylinositol (GPI)-anchor biosynthesis pathway is also upregulated in high-genomic instability samples (Figure 2F). The enhancement of GPI-anchor biosynthesis pathway activity could recruit macrophages to tumor tissues to generate TAM polarization (Dangaj et al., 2011), suggesting that the high tumor invasion and metastasis ability shown by high-genomic instability samples may be caused by the upregulation of GPI-anchored protein. All these indicate that the reprogramming of metabolic pathways provides the necessary preparations for tumor proliferation and invasion and is also the basis for tumor heterogeneity.

### Mutated LncRNA Stimulates Tumor Progression

**Figure 3:**
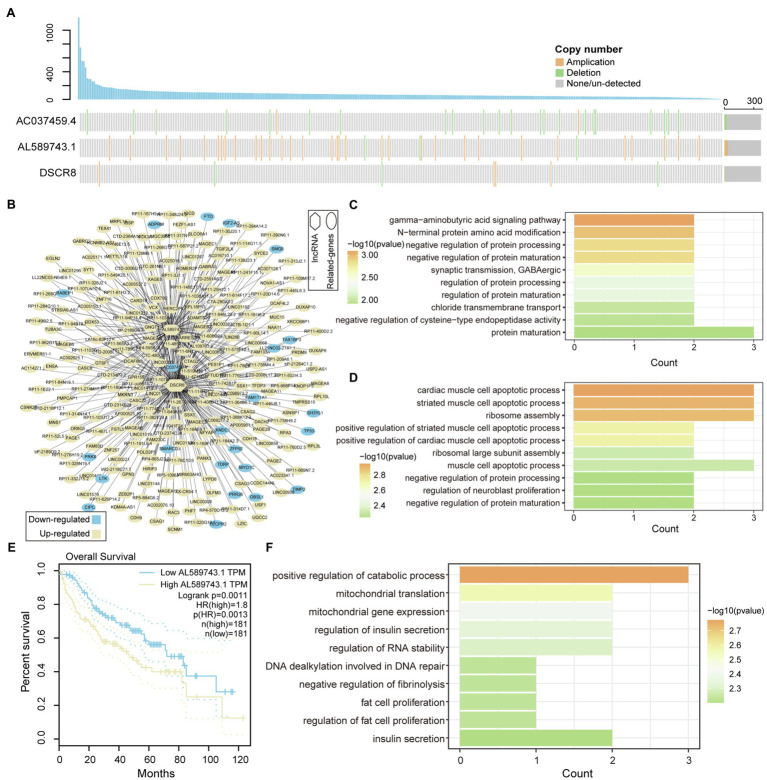
Functional identification of mutation-driven lncRNA. **(A)** Waterfall plot illustrates variation types of copy number (amplification, deletion, and none/un-detected) for each sample on lncRNA, including AC037459.4, AL589743.1, and DSCR8. The up-panel shows the number of mutated genes in each sample. **(B)** The relationship between mutation-driven lncRNA and co-expressed genes is shown by network. Circles represent co-expressed genes with lncRNAs; squares represented mutation-driven lncRNAs. Upregulated genes are marked with yellow; downregulated genes are marked with blue. **(C,D)** The bar graphs show the GO function enrichment results for genes co-expressed with lncRNA AC037459.4 and AL589743.1. **(E,F)** Survival difference between the two groups of samples with high and low expression of AL589743.1 and DSCR8. Univariate cox regression algorithm and log-rank test are used to evaluate the relationship between the expression of Md-lncRNA and patient survival.

LncRNA has become an important participant in almost every level of gene function and regulation (Qian et al., 2019; Wang et al., 2021). It is intriguing to identify the driver mutation lncRNA between high- and low-genomic instability samples. We extracted lncRNAs that were significantly differentially expressed between high- and low-genomic instability samples based on lncRNA annotation data, and combined CNV and somatic mutation data to identify three Md-lncRNAs (Figure 3A). We found that samples with Md-lncRNA *AL589743.1* copy number amplification clustered in highly mutant samples. Next, the Pearson correlation algorithm was used to identify DEGs that are significantly related to these three Md-lncRNAs at the gene expression level. We found that 412 DEGs (value of *p*<0.01 and correlation coefficient |R| >0.3) are involved in the regulatory network co-expressed with these three Md-lncRNAs (Figure 3B). To identify the role of these three mutation-driven lncRNAs in the carcinogenic mechanism of LIHC, gene ontology (GO) was used to perform functional enrichment analysis on DEGs that are significantly related to these three mutation-driven lncRNAs. We found that DEGs co-expressed with Md-lncRNA *AC037459.4* are mainly involved in the fat metabolism process of liver tissue (Figure 3C). The abnormal fat metabolism was the key cause of fatty liver, liver cirrhosis, and even liver cancer (Alves-Bezerra and Cohen, 2017). DEGs significantly related to lncRNA *AL589743.1* were enriched in protein processing and modification functional nodes (Figure 3D), suggesting that *AL589743.1* is involved in carcinogenic mechanisms by regulating the structure and function of proteins. We also found that the high expression of *AL589743.1* was significantly associated with poor patient’s prognosis (Figure 3E), indicating that *AL589743.1* can be used as an important target for the treatment of patients with advanced liver cancer. Further, DEGs co-expressed with Md-lncRNA *DSCR8* are mainly involved in protein processing and muscle cell apoptosis (Figure 3F). In previous studies, it has been confirmed that *DSCR8* can act as a miRNA sponge to activate the Wnt/β-catenin signaling pathway and promote the progress of LIHC (Wang et al., 2018). Taken together, all these results reveal that three Md-lncRNAs to promote tumor progression by participating in specific carcinogenic mechanisms.

### Prognostic Markers Correlated to LIHC

**Figure 4:**
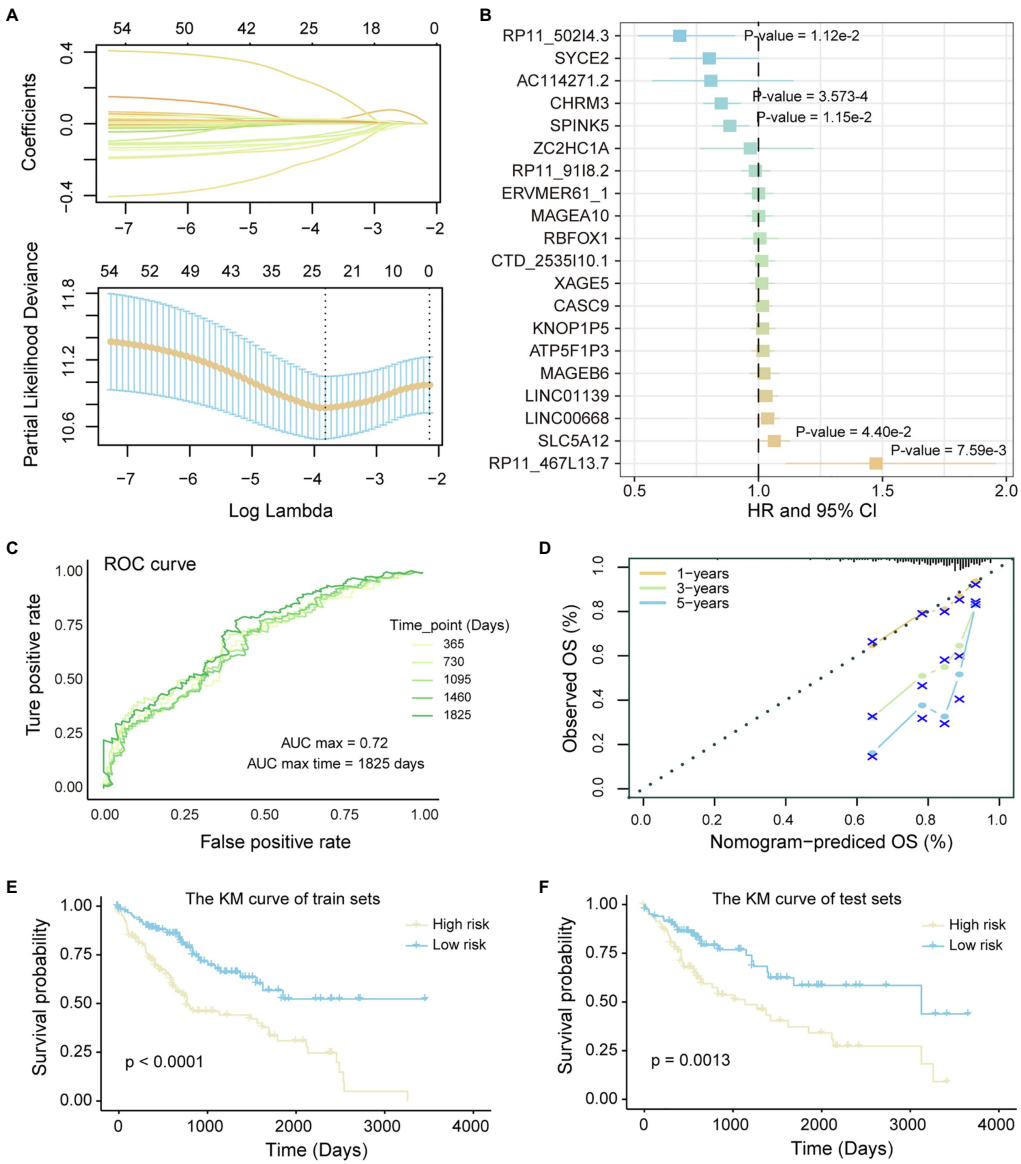
Identification of potential prognostic markers of LIHC. **(A)** Lasso regression model screen genes related to overall survival (OS) of LIHC patients. Variation curve of regression coefficient and λ value is shown. **(B)** COX risk regression to assess the association between the expression level of genes and patient survival. Genes that are significantly related to patient survival are added value of *p*. **(C)** The ROC curve reflects the predictive power of the risk regression model at five time points from 1 to 5 years. The different colored curves represent specific time points. **(D)** Calibration curve of nomogram. **(E,F)** Kaplan–Meier (KM) curves for survival of train set and test set in high- and low-risk groups. Log-rank test was used to calculate statistical significance.

LncRNA and transcripts co-expressed with it play an important role in the carcinogenic mechanism, which can be used as candidate prognostic markers. To identify prognostic markers of LICH, we first performed univariate cox regression and lasso regression algorithm to identify genes associated with OS in LIHC patients (see method). Then, 20 genes were identified and significantly correlated with the patient’s OS of LIHC (Figure 4A). Through the multivariate Cox regression constructed by the 20 genes and training set, five of which, *RP11-502I4.3, SPINK5, CHRM3, SLC5A12*, and *RP11-467L13.7*, were identified as prognostic markers for LIHC (Figure 4B; Supplementary Figure S2). To evaluate the predictive performance of the model, we showed the prediction results using ROC for five time points. We found that the risk prediction result reached the maximum AUC value of 0.72 (Figure 4C). Further, the nomograms algorithm was used to build a survival risk prediction model for LIHC (Supplementary Figure S3). The calibration curve was also used to validate the stability of the risk prediction model (Figure 4D). Moreover, the risk scoring model was constructed as follows: risk score=−0.37*RP11-502I4.3–0.11*SPINK5–0.16*CHRM3 +0.06*SLC5A12 +0.42*RP11-467L13.7. The samples of train set and test set were, respectively, divided into high- and low-risk groups based on the median risk score. We found that high-risk samples in train set are associated with poor prognosis of LIHC patients (Figure 4E). The test set also showed the same prediction results as the train set (Figure 4F), indicating the reliability of the risk score model in predicting the prognostic risk of patients. Taken together, we have identified five potential prognostic markers in LIHC, which can be used for clinical diagnosis.

### Tumor Progression Regulated by the Immune Microenvironment

**Figure 5:**
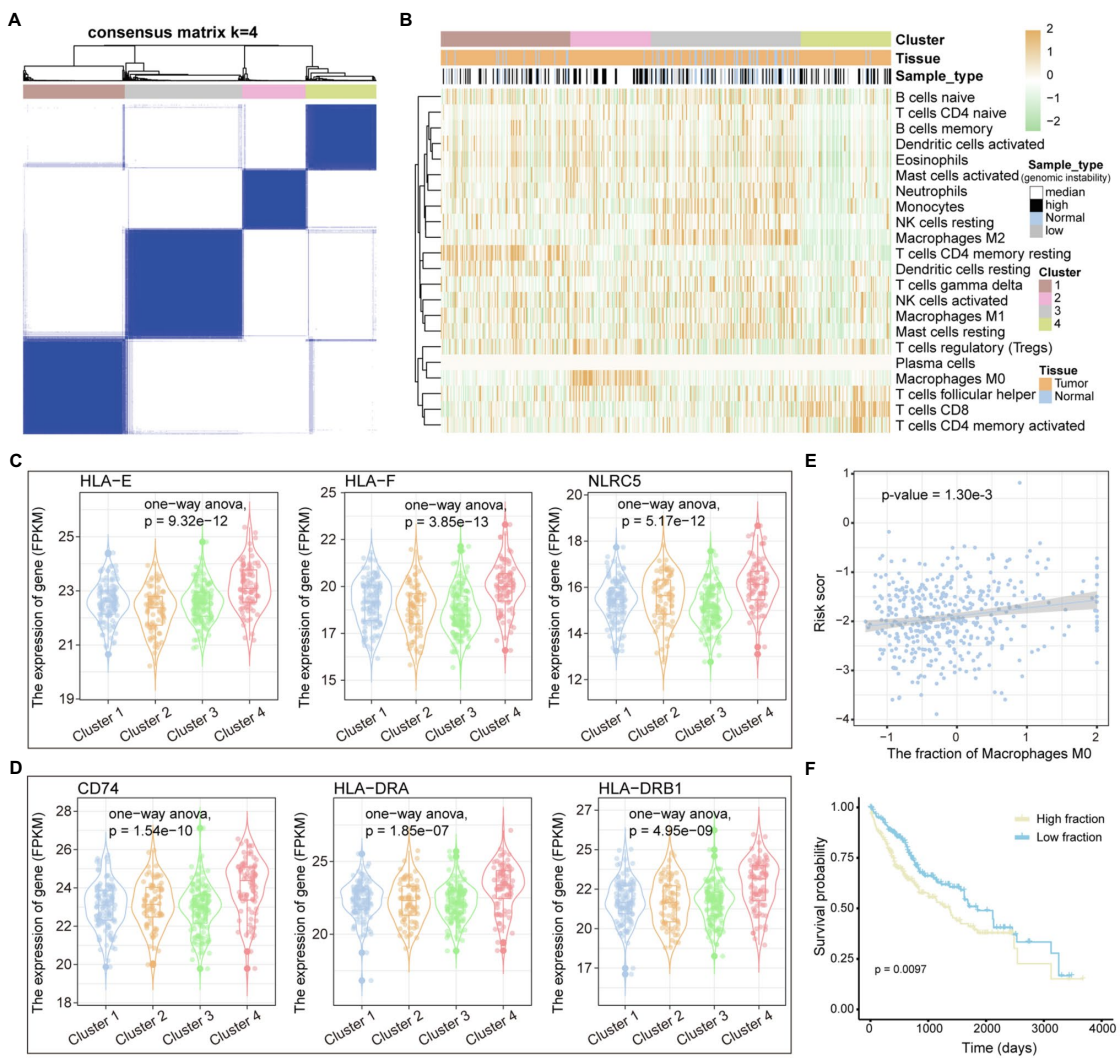
Immune cell components of LIHC patients. **(A)** The samples are divided into several clusters based on the immune cell components of each sample. The consistency matrix is drawn as a heat map, and the column labels show the clusters. **(B)** The 22 immune cell fractions of each sample were displayed by heat map. Column labels, including clusters, tissue origin, and type of variation of samples, are displayed. **(C,D)** The relationship between genes encoding MHC I and MHC II molecules and clusters defined by immune cell components is shown by boxplot. ANOVA is used to calculate statistical significance. **(E)** The correlation between the fraction of macrophages M0 and the risk score is shown. **(F)** KM curves for survival in high and low fraction groups of macrophages M0. Log-rank test is used to calculate statistical significance.

The tumor immune microenvironment plays an important role in the occurrence and development of tumors (Lei et al., 2020). The remodeling of the immune microenvironment is conducive to the progress of the tumor (Hinshaw and Shevde, 2019). Therefore, we used the CIBERSORTx tool to calculate the immune cell abundance of each LIHC sample and paracancerous tissue sample through the deconvolution algorithm that is a special kind of forward convolution, where the size of the input image is first enlarged by complementing the 0 at a certain scale, followed by rotating the convolution kernel and then forward convolution. For the 22 immune cell fraction matrices obtained, the consensus clustering algorithm was used to identify the immune subtypes of LIHC. We have defined four reliable tumor immune subtypes (Figure 5A and Supplementary Figure S4), which have specific immune cell composition. We found that the normal samples are mainly clustered in the third cluster, which has a relatively low content of CD8+ T cell and CD4+ T cell (Figure 5B). Multiple tumor samples have similar immune cell composition to normal samples in the third cluster, indicating that these samples are in immunosuppressed state. Different from other clusters, the fourth cluster of tumor samples has a higher content of CD8+ T cells (Figure 5B), suggesting that this type of LIHC patients is more suitable for immuno-targeted therapy. In order to explore the formation mechanism of tumor immunosuppressive microenvironment, we calculated the content of major histocompatibility complex (MHC). We found that genes involved in the synthesis of *MHC-I* have lower expression levels in the third cluster and significantly higher expression in the fourth cluster (Figure 5C), indicating that the immunosuppression of the third cluster may be caused by the loss of tumor immunogenicity. The *MHC-II* molecule, which is the CD4+ T-cell binding partner (Marty Pyke et al., 2018), also had lower expression level in the third cluster (Figure 5D). Next, by linking the immune cell fraction and risk score of each sample, we found that the fraction of Macrophages M0 is significantly related to the patient’s prognostic risk (Figure 5E). Tumor samples were divided into two categories (high/low fraction) based on the median of macrophages M0 fraction; we found that high-fraction samples are associated with poor patient’s prognosis (Figure 5F), suggesting that macrophages cells can promote tumor progression in the tumor microenvironment. Taken together, all these indicate that the loss of immunogenicity is a key factor for the formation of immunosuppressive microenvironment in multiple patients of LIHC.

## Discussion

In this study, we have integrated multi-omics data to reveal mutation-driven pathogenesis and immune landscape of LIHC. Through the statistics of the mutation location and type, we found the mutation characteristics of LIHC and defined two types of samples (high/low-genomic instability). We found that the inflammatory response was significantly enriched in the downregulated genes of the high-genomic instability samples by GSEA. Metabolic pathway activity analysis has shown that pyrimidine synthesis and GPI-anchor biosynthesis pathway are closely related to tumor progression and have low activity scores in high-genomic instability samples. We identified three mutations driving lncRNA and defined the molecular functions of these three mutations driving lncRNA in LIHC by constructing a co-expression network. Further, based on the genes involved in the co-expression network, we identified four prognostic markers, including *RP11-502I4.3, SPINK5, CHRM3, SLC5A12*, and *RP11-467L13.7*, through univariate cox regression and lasso algorithm screening. We also built risk score model to assess the prognostic risk of LIHC patients. Through the analysis of the immune cell fraction of tumor and paracancerous tissue samples, we defined four immune subtypes and found that the samples of immunosuppressive subtypes have low immunogenicity.

LIHC is a primary malignancy of the liver (Huang et al., 2016). Numerous of studies have tried to reveal the pathogenesis of LIHC and find effective treatments. For example, studies have shown that fibrosis of liver cells plays a vital role in the pathogenesis of liver cirrhosis and hepatocellular carcinoma (Liu et al., 2020). *TXNIP* activates the expression of oncogenes to inhibit the proliferation of hepatocellular carcinoma cells and induces apoptosis (Liu et al., 2017). In the last decade, the immune microenvironment of tumor has been a popular area of cancer biology research in relation to therapeutic targets for drug discovery. Although checkpoint inhibitors have been successfully used in cancer treatment, they are only effective in 10–40% of cases (Hamid et al., 2013; Callahan et al., 2014). Previous study has shown that checkpoint inhibitors do not trigger cancer-specific T-cell responses in some patients (Sharma and Allison, 2015). Therefore, it is necessary to reveal the relationship between the immune microenvironment of LIHC and tumor progression and the relationship between immune cells, which can be used to guide the combination medication of liver cancer patients.

Recent reports from developed countries indicate that metabolic disorders caused by diabetes, obesity, and fatty liver are risk factors for LIHC (Makarova-Rusher et al., 2016). Besides, the experimentally confirmed carcinogenic and regulatory mechanisms of lncRNA have been widely revealed (Wang et al., 2019). Genes related to lncRNA *AC037459.4* were identified involved in the fat metabolism process of liver tissue, suggesting that *AC037459.4* may mediate dysregulation of fat metabolism pathways in patients. Based on previous research on the identification of cancer prognostic markers (Yu et al., 2019), we identified five potential prognostic markers by multivariate Cox regression analysis, which can be used in the clinical diagnosis of patients and guiding their treatment. The subtype of LIHC with strong immunogenicity suggests that immune checkpoint inhibitor may have a better effect on these patients. The fraction of macrophages in tumor tissue was found to be significantly associated with the risk of death in patients, consistent with previous studies demonstrating the involvement of macrophages in tumor invasion and metastasis (Chen et al., 2020).

In conclusion, this study provided a mutation-driven metabolic landscape and immune landscape of LIHC. Three mutated lncRNAs were identified to drive transcriptional perturbed oncogenic pathways and affect patient prognosis. Five gene signatures associated with patient prognosis were identified through Cox regression and lasso regression. We also identified four immune subtypes for LIHC. In conclusion, all these findings provide theoretical guidance for the optimization of LIHC treatment strategies.

## Data Availability Statement

The original contributions presented in the study are included in the article/Supplementary Material, further inquiries can be directed to the corresponding author.

## Author Contributions

HeW, WJ, HaW, and PH conceived and designed the experiments. ZW, HL, and HY analyzed the data. HeW and WJ collected the data. HeW, WJ, and HaW validated the method and data. HeW and PH wrote this manuscript. All authors read and approved the final manuscript.

## Funding

This work was supported by the National Natural Science Foundation of China [81803010].

## Conflict of Interest

The authors declare that the research was conducted in the absence of any commercial or financial relationships that could be construed as a potential conflict of interest.

## Publisher’s Note

All claims expressed in this article are solely those of the authors and do not necessarily represent those of their affiliated organizations, or those of the publisher, the editors and the reviewers. Any product that may be evaluated in this article, or claim that may be made by its manufacturer, is not guaranteed or endorsed by the publisher.

## Footnotes

^1^ https://xenabrowser.net/

^2^ http://software.broadinstitute.org/gsea/msigdb

^3^ https://www.gencodegenes.org/

^4^ https://cibersortx.stanford.edu/

^5^ http://gepia2.cancer-pku.cn/#index
