# Supplementary material for: Identification of Mutation Landscape and Immune Cell Component for Liver Hepatocellular Carcinoma Highlights Potential Therapeutic Targets and Prognostic Markers
Source: Front Genet. 2021 Sep 16;12:737965. doi: 10.3389/fgene.2021.737965 (PMC8481807; doi:10.3389/fgene.2021.737965)
Supplement: Supplementary file 1 [file Data_Sheet_1.docx]

**Supplementary Figure**

**
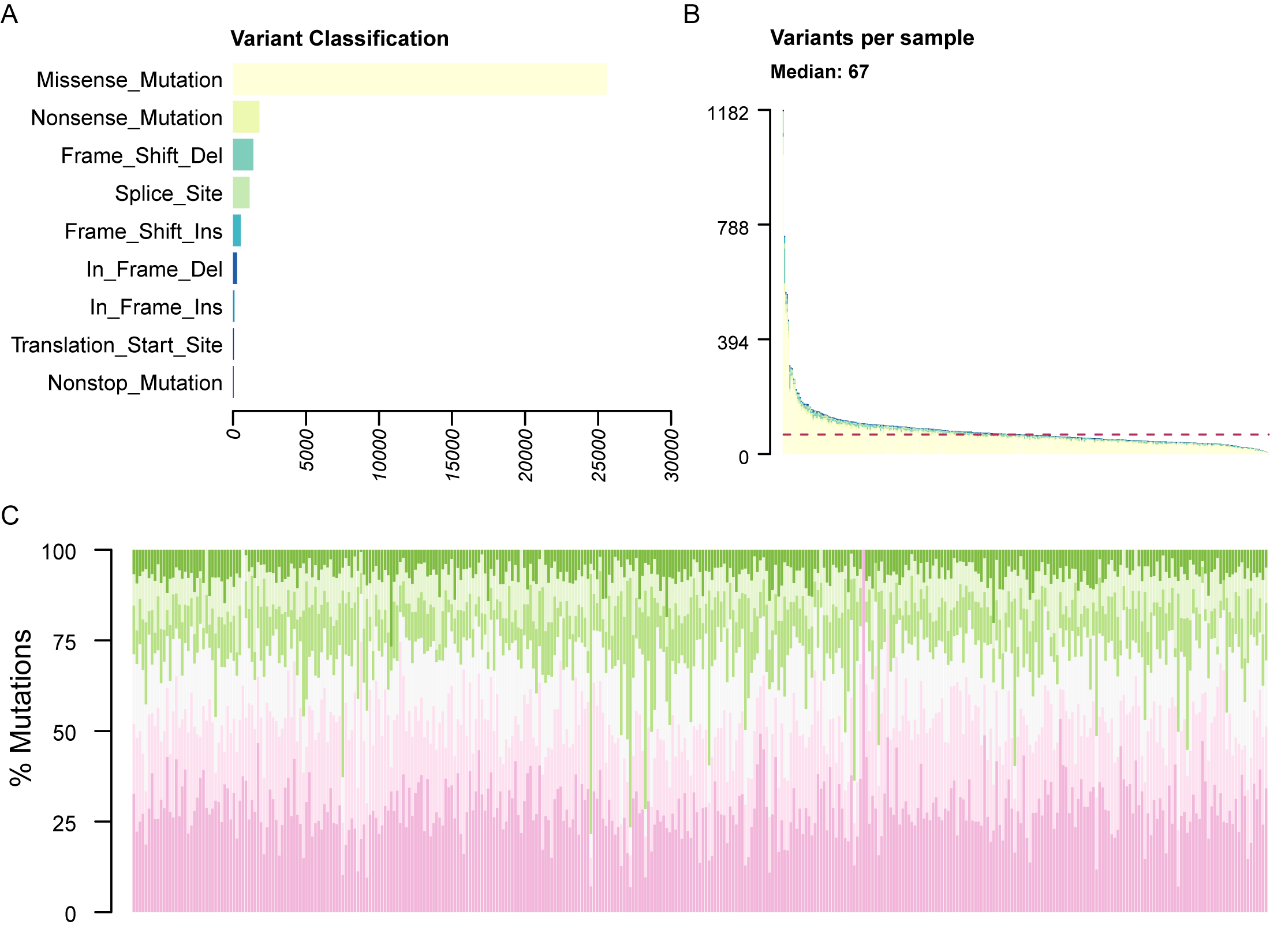
**

**Figure S1. (Related to Figure 1)** (A) Somatic mutations were classified into nine clusters according to function and location. The bar plot shows the number of mutations in each cluster. (B) The number of somatic mutations in each tumor sample was displayed as a bar graph. (C) The proportion of six types of base substitutions (including C>T, C>A, T>C, T>A, C>G, and T>G) in each tumor sample was shown by bar plot.


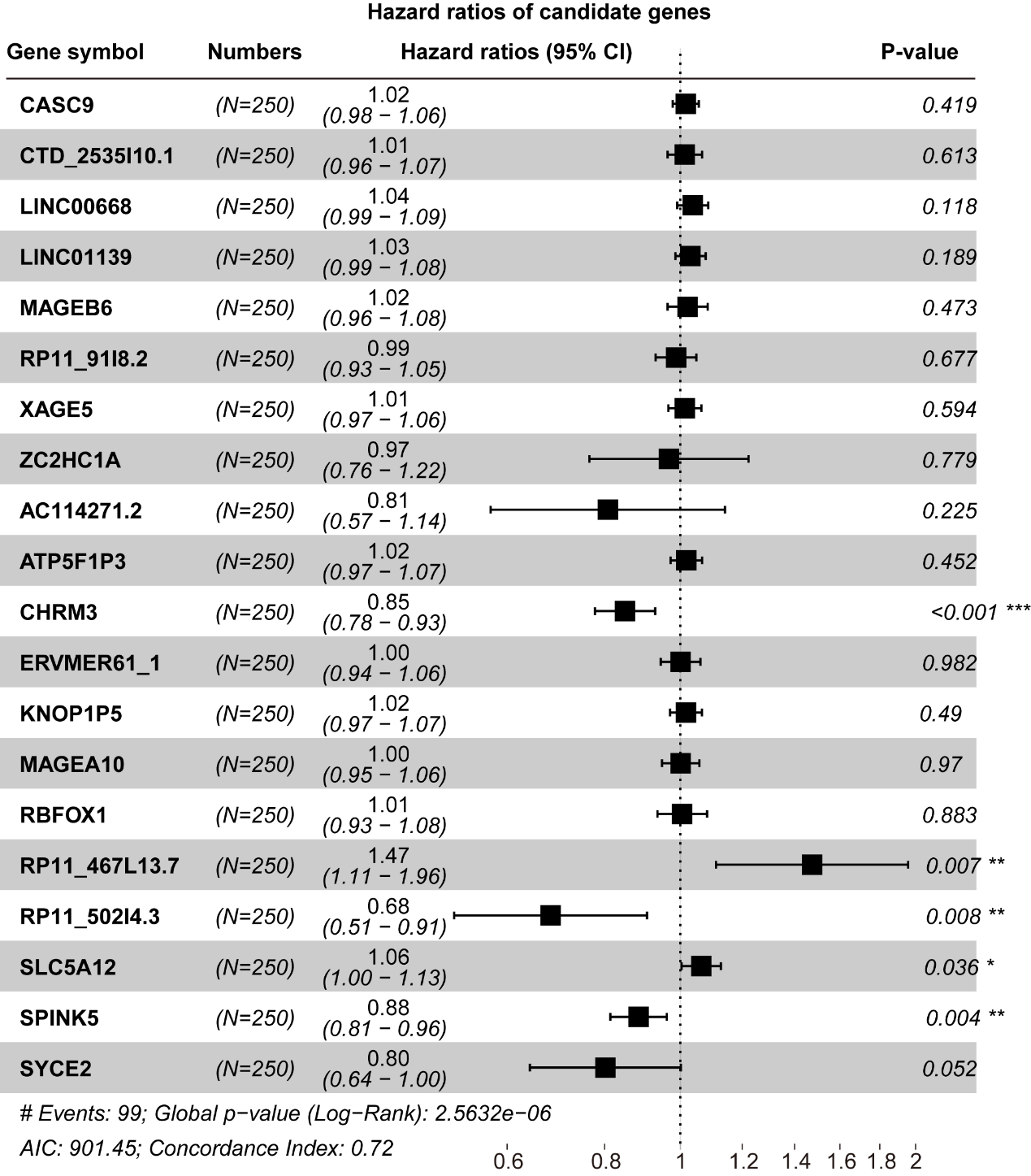


**Figure S2. (Related to Figure 4)** Forest plots for multivariate Cox risk regression models.


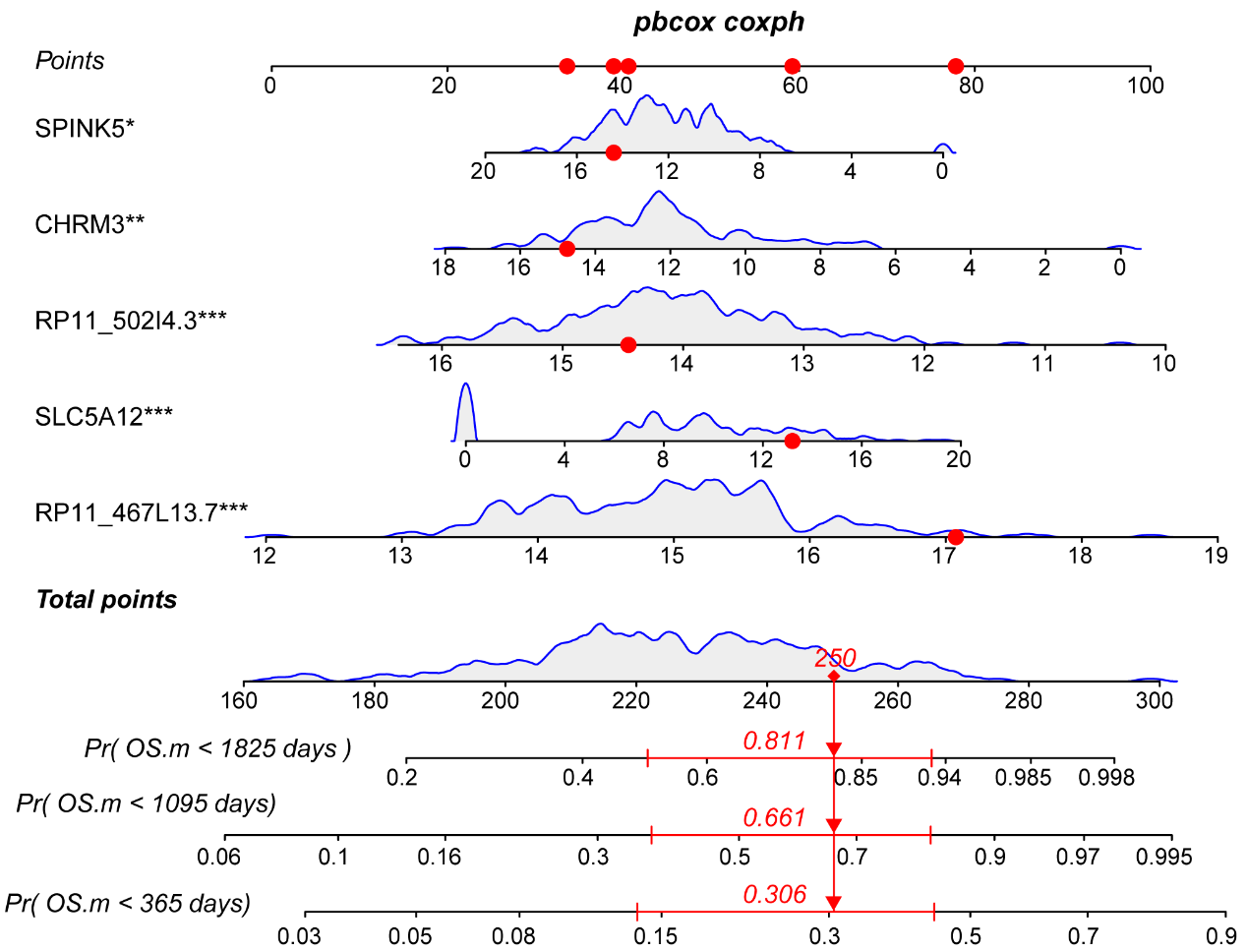


**Figure S3. (Related to Figure 4)** Nomogram for survival risk prediction of 365 (1 year), 1095 (3 years) and 1825 (5 years) days. The graph included five prognostic marker genes.


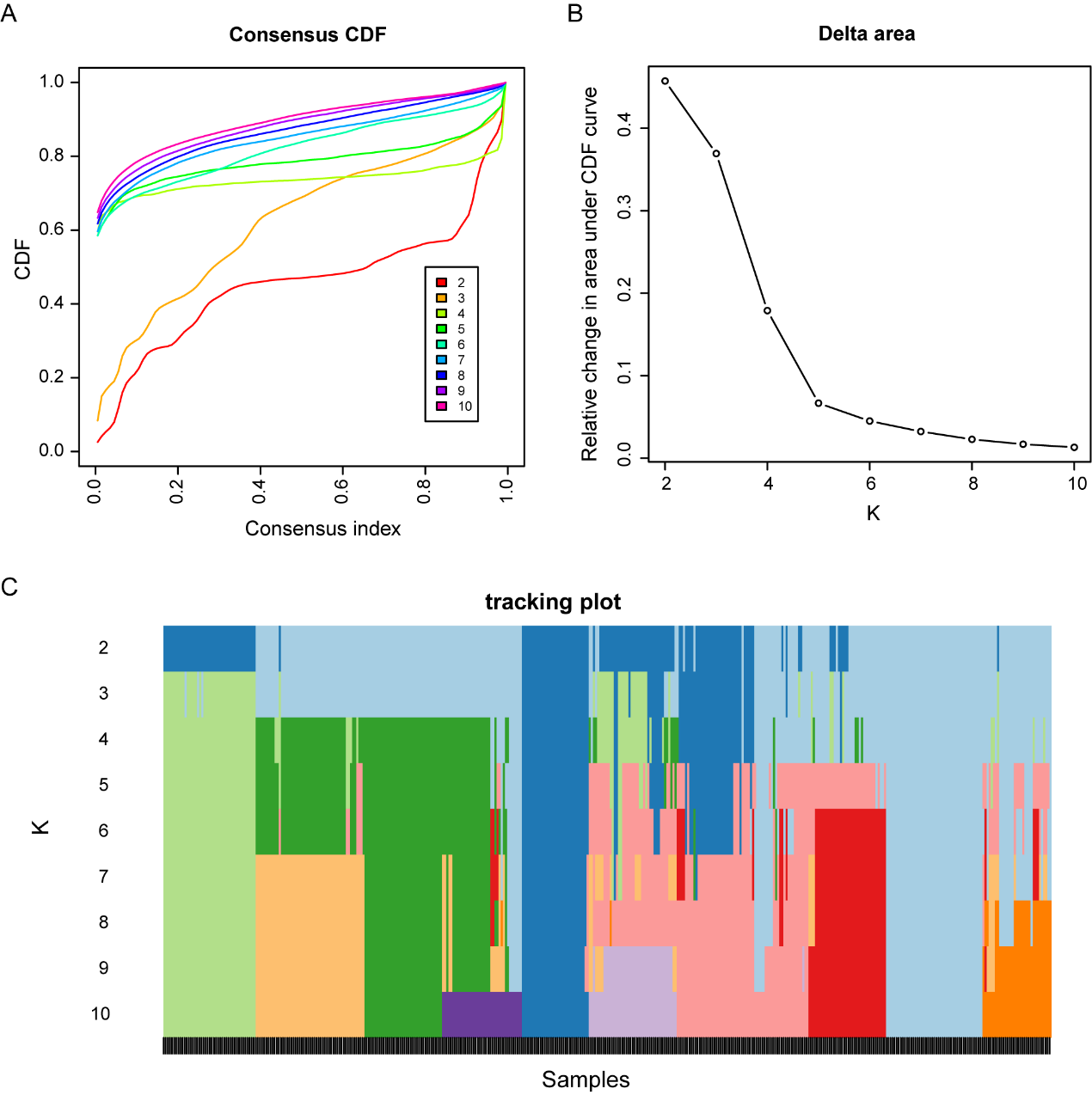


**Figure S4. (Related to Figure 5)** (A) This plot described the variation curve of the CDF with the consensus index as K, which determines the number of clusters, is varied from 2 to 10. (B) The delta area score (y-axis) indicates the relative increase in cluster stability. (C) This trajectory graph reflects the change in the cluster to which each sample belongs as K moves from 2 to 10.
